# Is Realising Evidence‐Based X the Future of Evidence Synthesis?

**DOI:** 10.1002/cl2.70037

**Published:** 2025-03-20

**Authors:** Gavin Stewart

**Affiliations:** ^1^ Evidence Synthesis Group, University of Newcastle upon Tyne Morpeth UK

Evidence synthesis (including systematic reviews and meta‐analysis) has a long evolution and has had major impacts across the sciences (Shadish and Lecy 2015), underpinning evidence‐informed decision‐making, particularly in specific health and social science domains. There has also been some penetration into environmental and climate sciences, but it is not [yet] the primary mechanism for science‐policy translation outside health and social sciences. Many methodologists have worked across health or social science domains, and there has long been a realisation that methods harmonisation is beneficial, seen in closer working between Cochrane and Campbell, and the formation of the Society for Research Synthesis methods to foster interdisciplinary learning cf (Stewart and Schmid 2015). Concurrently, the scope of applications has widened considerably, perhaps best exemplified by the global SDG synthesis coalition, who envisage robust evidence synthesis underpinning decisions made across all the sustainable development goals, targets and indicators. This would represent the full realisation of evidence‐based X, not evidence‐based health or social science or environment – but fully developed generic methodologies applicable irrespective of domain (EBX). To those of us who believe in generic methodologies and the need for coherent decision‐making across increasingly complex decision space, this evolution of evidence synthesis is long overdue, but it is not without challenges.

Perhaps the three largest barriers to overcome are the plethora of untrustworthy evidence in our publications and databases, lack of coherent whole systems thinking and the difficulties of developing pipelines for methods innovation that maintain rigour.

## 1 Untrustworthy Evidence

The unpalatable truth that a large fraction (arguably, even the largest fraction) of scientific publications are somewhere between misleading and downright wrong is horrifying and contested by most scientists who are not research methodologists. Most believe that peer review and our publication procedures are adequate to safeguard scientific integrity. They are quite simply wrong! Doug Altman's seminal paper on the scandal of poor medical research from 1994 could be written today in any domain of applied science (Altman 1994). In 2005, John Ioannidis argued that ‘Most published research findings are false’, particularly in fields with large numbers of researchers exploring small effects (Ioannidis 2005). Richard Horton was scathing about progress in 2015 ‘Much of the scientific literature, perhaps half, may simply be untrue. Afflicted by studies with small sample sizes, tiny effects, invalid exploratory analyses, and flagrant conflicts of interest, together with an obsession for pursuing fashionable trends of dubious importance, science has taken a turn towards darkness’. He warned, ‘poor methods get results’ (Horton 2015). There has been some progress, particularly in the field of psychology, with the realisation that questionable research practices were resulting in a ‘replication crisis’ and the subsequent development of the ‘open science’ movement to remedy this, but the problems with evidence remain prevalent. This has important ramifications for EBX.

First, scientists need to devote considerable efforts to remedying this situation to ensure that the foundational primary studies are fit for purpose. This will be particularly challenging in domains unfamiliar with formalised evidence synthesis where the need to ensure the integrity of scientific data may not be fully accepted or understood. Endeavours such as Entrust‐PE (O'Connell et al. 2024) could be generalised to achieve this. Entrust‐PE is an international, interdisciplinary network established to develop an integrated framework for enhancing and facilitating the trustworthiness of pain research by engaging with patients, authors, clinicians, scientists, publishers and funders of science. The primary focus of Entrust‐PE is on the conduct of research, but initiatives to develop and promote adherence to reporting standards and the generation of core common outcome sets will also be necessary. Making sure that scientists measure the right things, the right way and report their studies properly is a huge but potentially transformative task.

Second, the important roles of evidence synthesis in critically appraising study validity and identifying divergent data must remain central pillars of robust synthesis despite the resource‐intensive nature of this work. Effect sizes speak the truth where hacked and harked *p* values and words do not – as long as they are recalculated, evaluated and contextualised, considering confounders, heterogeneity and publication biases. There are no shortcuts to thoughtful synthesis, be it quantitative or qualitative! This is demanding and skilled work, even with simple outcomes and randomised controlled trials. The high heterogeneity‐multiple study design syntheses that comprise most evidence relevant to broader sustainable development goals are much more challenging, requiring a structured appraisal to identify and highlight the massive uncertainties in evidence so often obscured by primary studies, overhyped summaries or conveniently compliant policy briefs.

## 2 Systems Thinking

The need to embed evidence synthesis in broader systems to develop effective action in complex contexts or to consider broad policy questions has long been recognised as challenging. The medical model of parameterising health economic models with meta‐analysis has served health technology assessment well generally, but systems boundaries are tightly defined in that context, and even there, uncertainties often relate to implementation utilities that are imprecisely known rather than the effects of interventions resulting in incoherence. Beyond that, decision and systems modellers rarely work with meta‐analysts, and the qualitative worlds of logic maps rarely meet the quantitative worlds of directed acyclical graphs or Bayesian decision models. Clearly, there is huge scope to ascertain how existing tools can be integrated and develop new ways of thinking in this space to support policy makers in making broad policy decisions. The idea of developing suites of reviews or meta‐analyses to underpin one decision has been little explored, although large network meta‐analyses can replace many meta‐analyses of pairwise comparisons, which is heuristically similar. For example, Birkinshaw et al. (2023) undertook a network meta‐analysis of 25 antidepressants across 3 pain conditions in a unified analysis that would traditionally have required > 100 pairwise systematic reviews, none of which would have determined which antidepressant should be the top‐ranked treatment for pain. Utilising such methods would also require potentially new ways of working with the policy community to define decision options, utilities and systems boundaries but could effectively direct research to minimising important uncertainties as well as provide mechanisms to support transparent and coherent policy making.

## 3 Methods Innovation

Every methodologist will have their own view of research priorities relevant to EBX. The scope is vast, ranging from developing guidance on mixed methods synthesis to generating critical appraisal tools that flag concerns across study designs to expressing uncertainties using threshold analyses or exploring heterogeneity with model averaging routines. Two areas of methodological innovation driven by the need to make broad policy decisions rapidly, namely systematic mapping and rapid evidence assessment, require particularly careful consideration.

Breadth is often provided by evidence and gap maps, or topic models generated either using traditional systematic review methods or increasingly artificial intelligence. Such maps provide useful overviews of what evidence is available but critically do not inform policy directly. Scientists have a tendency to pontificate in inverse relation to knowledge, thus twenty studies linking two nodes could have less evidential value than one good study. The temptation to utilise such maps without principled synthesis should be resisted. Berrang‐Ford et al. (2021) demonstrate the utility of systematic maps in a principled manner – using AI to generate a large‐scale overview – with multiple subsequent systematic reviews used to inform policy.

A plethora of methodologies, synthesis products and terminology also surround ‘rapid review’. The term has become meaningless, incorporating everything from undertaking a full systematic review rapidly to abandoning every methodological expectation of a full review to expedite and simplify evidence acquisition and synthesis. Clearly, the latter represent a dangerous, misleading form of research waste despite their prevalence in some domains. Perhaps the most invidious and unjustifiable form of synthesis – vote counting is often employed in this context. The safest course of action for a policy maker presented with evidence generated in this manner is to put it in the bin – whether generated by a human or a machine. Note that this is not to say that good rapid reviews do not exist. Judicious use of shortcuts to expedite rapid synthesis are perfectly germane, provided they transparently communicate the uncertainties inherent in their methods and consider the impact on review findings. Campbell and other coordinating bodies, provide good guidance on using these methods appropriately.

Addressing the systemic problems with the generation and synthesis of scientific information may feel intractable, but as authors, reviewers and editors – commissioners or users of science – we can all do our bit. Essentially, do what you can to avoid research waste! Make sure your research is useful and useable and, above all, transparent. Campbell Collaboration authors are well to fore in this endeavour and should be proud of their achievments. The broader challenges in realising the full potential of EBX are non‐trivial but so is the reward. The dream of rational decision‐making underpinned by a coherent and reliable evidence architecture across all science domains is perhaps closer than ever. Given the incoherence of our current science pipelines and the post‐truth political environment, the need for it may also be greater than ever.
